# Postoperative imaging of the intestinal microcirculation

**DOI:** 10.1186/cc12147

**Published:** 2013-03-19

**Authors:** I Abdo, R Hall, D Henzler, V Cerny, CH Lehmann

**Affiliations:** 1 Dalhousie University, Halifax, Canada; 2 Charles University, Medical Faculty in Hradec Kralove, Czech Republic

## Introduction

Sidestream dark-field (SDF) imaging was introduced recently to study the sublingual microcirculation in humans. Patients with ileostomies offer a unique access to the intestinal microcirculation. To date, no reference ranges for standard microcirculatory parameters of the gut are available. Therefore, the aim of our study was to establish a database for postoperative microcirculatory parameters for ileostomies.

## Methods

For this observational prospective cohort study 77 patients were screened. In total, 165 SDF measurements could be obtained. All patients included had bowel surgery for chronic inflammatory bowel disease or intestinal malignancies and received an ileostomy. Patients were excluded if they had signs of local infection or bleeding, or if they were admitted to the ICU for sepsis or perioperative complications. The SDF device was gently inserted into the stoma at a depth of 3 to 4 cm, and five real-time images were recorded. All videos recorded were analyzed offline using AVA^® ^software (Microvision Medical, Amsterdam, the Netherlands). The following parameters were quantified: microvascular flow index (MFI), total vessel density (TVD), perfused vessel density (PVD), and proportion of perfused vessels (PPV). Patients were followed for 3 days post surgery with five images captured every day.

## Results

We were able to capture clear images of the small intestine microvasculature. Distinct villi were visible with a dense network of microvessels (diameter: 6 to 17 μm). Mean TVD (± 2SD) was 19.3 (± 1.0) mm/mm^2^, PVD: 18.5 (± 1.1) mm/mm^2^, PPV: 94.5 (± 5)% and MFI: 2.8 (± 0.1). Patients' age, sex and comorbidity had no significant impact on postoperative microvascular parameters. No significant changes of the microvascular parameters were observed during the first 3 postoperative days.

## Conclusion

SDF imaging is a feasible, non-invasive bedside method to study the postoperative intestinal microcirculation. The established reference ranges are useful for early detection of postoperative local complications and studies of microcirculatory changes induced by systemic pathologies; for example, in sepsis.

